# FAM96A Protects Mice From Dextran Sulfate Sodium (DSS)-Induced Colitis by Preventing Microbial Dysbiosis

**DOI:** 10.3389/fcimb.2019.00381

**Published:** 2019-11-18

**Authors:** Ang Yin, Yang Luo, Wei Chen, Minwei He, Jin Hai Deng, Ning Zhao, Lulu Cao, Lu Wang

**Affiliations:** ^1^Department of Immunology, Center for Human Disease Genomics, Health Science Center, School of Basic Medical Sciences, Peking University, Beijing, China; ^2^Key Laboratory of Medical Immunology, School of Basic Medical Science, Peking University, Ministry of Health, Beijing, China

**Keywords:** FAM96A, inflammatory bowel diseases, gut microbiota, DSS, intestinal mucosa

## Abstract

Family with sequence similarity 96 member A (FAM96A) is an evolutionarily conserved intracellular protein that is involved in the maturation of the Fe/S protein, iron regulatory protein 1 (IRP1), and the mitochondria-related apoptosis of gastrointestinal stromal tumor cells. In this study, we used a mouse model of chemically induced colitis to investigate the physiological role of FAM96A in intestinal homeostasis and inflammation. At baseline, colons from *Fam96a*^−/−^ mice exhibited microbial dysbiosis, dysregulated epithelial cell turnover, an increased number of goblet cells, and disordered tight junctions with functional deficits affecting intestinal permeability. After cohousing, the differences between wild-type and *Fam96a*^−/−^ colons were abrogated, suggesting that FAM96A affects colonic epithelial cells in a microbiota-dependent manner. *Fam96a* deficiency in mice resulted in increased susceptibility to dextran sulfate sodium (DSS)-induced colitis. Importantly, the colitogenic activity of *Fam96a*^−/−^ intestinal microbiota was transferable to wild-type littermate mice via fecal microbial transplantation (FMT), leading to exacerbation of DSS-induced colitis. Taken together, our data indicate that FAM96A helps to maintain colonic homeostasis and protect against DSS-induced colitis by preventing gut microbial dysbiosis. This study used gene knockout animals to help to understand the *in vivo* effects of the *Fam96a* gene for the first time and provides new evidence regarding host–microbiota interactions.

## Introduction

Inflammatory bowel disease (IBD) is a collection of chronic remittent inflammatory disorders that are associated with a variety of factors such as host genetics, the environment, and intestinal microbes (Wlodarska et al., 2015). IBD is prevalent in Western countries, affecting approximately 0.5% of the total population (Molodecky et al., 2012). It mainly consists of two clinically defined chronic disorders: Crohn's disease (CD) and ulcerative colitis (UC). Patients with IBD have a higher risk of colon cancer; about 7–8% of IBD patients eventually develop colon cancer within 20 years (Gillen et al., 1994). There are several animal models that help to understand IBD pathogenesis and to develop novel therapeutic approaches. Among these, dextran sulfate sodium (DSS) administration is a commonly used method, which can induce reproducible acute colitis characterized by bloody diarrhea, ulcerations, and leukocyte infiltration (Perše and Cerar, 2012). Although the mechanisms underlying IBD are not fully understood, many studies have indicated that the dysregulation of host–microbe interactions is a key contributor (Abraham and Medzhitov, 2011).

Trillions of commensal microorganisms inhabit in mammalian gastrointestinal tracts at extremely high densities. These communities, termed microbiotas or microbiomes, benefit the hosts in many ways, including improving digestion, preventing colonization by pathogenic microbes (Holmes et al., 2011), and assisting the development of competent innate and acquired mucosal immune systems (Macpherson and Harris, 2004). Recent research has revealed that the intestinal microbiota is closely associated with several inflammatory diseases, including IBD (Thomas, 2018). The decrease of beneficial strains like *Faecalibacterium prausnitzii* (Sokol et al., 2008) and/or increase of pathogenic strains such as entero-invasive *Escherichia coli* (Darfeuille-Michaud et al., 2004) promote IBD development. The commensal gut flora regulates the intestinal epithelium phenotype and the progression of colitis. For instance, germ-free mice exhibit increased intestinal permeability (Smith et al., 2007), reduced intestinal epithelial cell (IEC) proliferation, migration, and renewal (Rakoff-Nahoum et al., 2015), and decreased numbers of Paneth and goblet cells (Yu et al., 2016). In addition, attaching and effacing (A/E) pathogens enterohaemorrhagic *E. coli* (EHEC), enteropathogenic *E. coli* (EPEC) and murine A/E pathogen *Citrobacter rodentium* disrupt multiple host tight junction (TJ) proteins *in vivo*, impairing the IEC barrier and increasing the risk of colitis (Guttman et al., 2006). The microbial composition is affected by many external factors such as obesity, diet, diseases, and host genetic factors, involving antimicrobial peptides (AMPs), IgA, and inflammasomes (Turnbaugh et al., 2006; Wen et al., 2008; Salzman et al., 2010; Elinav et al., 2011; Okai et al., 2016). Lack of *Il22, Nod2*, or *Nlrp6* in mice results in the transformation of the intestinal microbiota to a colitogenic set, and the transmission of this colitogenic microbiota increases susceptibility to colitis in wild type (WT) recipient mice (Elinav et al., 2011; Couturier-Maillard et al., 2013; Zenewicz et al., 2013).

Family with sequence similarity 96 member A (FAM96A) is a ubiquitously expressed and evolutionarily conserved protein that contains a domain of unknown function 59 (DUF59). The homology of FAM96A between *Homo sapiens* and *Mus musculus* is as high as 85%. FAM96A is a member of the cytosolic Fe/S protein assembly machinery and it regulates cellular iron homeostasis by regulating the maturation of iron regulatory protein 1 (IRP1) (Stehling et al., 2013). FAM96A also possesses apoptosome-activating potential and participates in suppressing tumor growth in gastrointestinal stromal tumor (GIST) cells (Schwamb et al., 2015). Although FAM96A exerts various functions, its role in regulating the gut microbiota and colitis remains unknown.

In this study, we used *Fam96a* knockout (KO) mice to investigate the regulatory role of *Fam96a* related to the colonic microbiota and susceptibility to colitis. *Fam96a* KO mice exhibited microbial dysbiosis, an altered colonic epithelium phenotype, and enhanced susceptibility to DSS-induced colitis. In addition, the altered IEC phenotype and the increased susceptibility to gut inflammation could be transferred to wild-type (WT) mice by transferring the *Fam96a*^−/−^ intestinal microbiota.

## Materials and Methods

### Mice

All mice used in this study had a C57BL6/J background. *Fam96a*^−/−^ mice were generated by the Model Animal Research Center of Nanjing University. *Fam96a*^*fln*/*fln*^ mice were crossed with Zp3-cre mice to obtain Zp3-cre-*Fam96a*^*flox*/−^ female mice, which were further crossed with male C57BL6/J mice to obtain *Fam96*^+/−^ mice. The heterozygote mice were crossed with each other to generate WT and *Fam96a*^−/−^ mice for the experiments. For the cohousing experiments, age- and gender-matched WT and *Fam96a*^−/−^ mice were cohoused at a 1:1 ratio for at least 8 weeks. All mice were housed under specific-pathogen-free (SPF) conditions at Peking University Health Science Center. All the experiments were carried out in accordance with guidelines approved by the Institutional Animal Care and Use Committee of Peking University.

### DSS-Induced Colitis

For the acute colitis experiments, age-matched (8–12 weeks) mice of the same gender were fed with water containing 2.5–3.0% (w/v) DSS (molecular weight: 36–50 kDa; MP Biomedicals, Santa Ana, CA, USA) for 5–9 days, and the mice were then euthanized at indicated time points. For the survival experiments, acute colitis was induced with 2.5% (w/v) DSS in the drinking water for 5 days followed by normal drinking water until the end of the experiment. Body weight, stool softness, and blood in the rectum or fur were recorded daily. Disease Activity Index (DAI) was calculated as previously described (Luo et al., 2016); the calculation involves three parameters: (a) diarrhea (0, normal; 2, loose stools; 4, watery diarrhea); (b) hematochezia (0, no bleeding; 2, slight bleeding; 4, gross bleeding); (c) percentage weight loss (0, none; 1, 1–5%; 2, 5–10%; 3, 10–20%; 4, >20%).

### Histologic and Immunohistochemistry Analysis

The colons were washed thoroughly with cold phosphate-buffered saline (PBS), fixed in 4% paraformaldehyde for at least 24 h and then embedded in paraffin. Subsequently, 4-μm-thick sections were stained with hematoxylin and eosin (HE) (ZSGB-BIO, Beijing, China) according to standard procedures. The colitis severity was measured in a blinded manner by a professional pathologist from the Department of Pathology at Peking University based on previously described double-blind methods (Li et al., 2015). Briefly, the histopathologic score was calculated by combining the score for (a) leukocyte infiltration (score of 0–3; 0, rare leukocytes in lamina propria; 1, increased leukocytes in the lamina propria; 2, confluence of leukocytes in the submucosal part of the colon; 3, transmural infiltration of leukocytes) and (b) tissue damage (score of 0–3; 0, normal tissue; 1, discrete focal lymphoepithelial lesions; 2, mucosal erosion/ulceration; 3, extensive mucosal damage and extension through deeper structures of the bowel wall). For the immunohistochemistry experiments, tissue slides were stained with anti-Mucin 2 (MUC2; GeneTex, Irvine, CA, USA) anti-Ki67 (Abcam, Cambridge, MA, USA), and the slides were then washed three times with PBS before incubation with secondary antibodies (ZSGB-BIO, Beijing, China). Signals were developed with 3,3′-diaminobenzidine (DAB; ZSGB-BIO, Beijing, China) in conjunction with a hematoxylin counterstain.

### Terminal Deoxynucleotidyl Transferase dUTP Nick End Labeling (TUNEL) Assay

Cell death was assessed by TUNEL assays of formalin-fixed paraffin-embedded slides using a TUNEL cell death detection kit (Beyotime Biotechnology, Beijing, China) according to the manufacturer's instructions, and 4′,6-diamidino-2-phenylindole (DAPI; Sigma, St. Louis, USA) was used to stain the nuclei.

### Goblet Cell Assay

To identify goblet cells, tissue sections were stained with Alcian blue/periodic acid–Schiff (AB-PAS) (Solarbio, Beijing, China) according to the manufacturer's instructions. The goblet cells were stained bluish violet. A hematoxylin counterstain was used to identify the nuclei.

### Colon Homogenization and Cytokine Detection

Freshly extracted 2-cm-long specimens from the distal colons were weighed and cut into small pieces (approximately 1 mm^2^), and the pieces were then placed in cold PBS with protease inhibitor cocktail (Roche, Penzberg, Germany). The samples were homogenized with a FastPrep-24 instrument (MP Biomedicals, Santa Ana, CA, USA). To remove the tissue particles, the homogenate was then centrifuged at 12,000 rpm. for 20 min at 4°C and the supernatant was collected. A LegendPlex inflammatory Cytokines Kit was used according to the manufacturer's instructions to detect the cytokine concentrations (in pg/mg tissue protein) in the supernatant.

### Intestinal Permeability

Mice were fasted for 8 h, and 400 μg fluorescein isothiocyanate (FITC)-dextran (4 kDa, Sigma-Aldrich, St. Louis, USA)/g body weight was administered by gavage. At 4 h later, blood was extracted from the inner canthus. The serum was harvested by clotting followed by centrifugation. FITC-dextran fluorescence in the serum samples was measured at an emission wavelength of 490 nm and an excitation wavelength of 520 nm. Serial dilutions of FITC-dextran ranging from 0 to 30 μg/ml were used as standards.

### Antibiotic Treatment and Fecal Microbial Transplantation (FMT)

The gut microbiota was depleted as previously reported (Guo et al., 2018) by treating with broad-spectrum antibiotics [ampicillin 1 g/L, metronidazole 1 g/L, neomycin sulfate 1 g/L, and vancomycin 500 mg/L (Solarbio, Beijing, China)] in the drinking water for 2 weeks. For the experiment used to verify the colitogenic role of gut microbiota from *Fam96a*^−/−^ mice, the FMT was performed as previously reported (Guo et al., 2018) with minor modifications following the antibiotic treatment. In brief, fresh feces were collected within 30 min and were immediately resuspended in cold PBS. The suspension was passed through a 70-μm mesh cloth to remove large particles and then administered to the recipient mice by oral gavage. The gavage was conducted daily for 2 weeks.

### Real-Time PCR for Microbiota Analysis

Fresh fecal pellets were collected and total DNA was immediately isolated using a Stool Genomic DNA Kit (CWBIO, Beijing, China) according to the manufacturer's instructions. The fecal samples were stored at −80°C if they could not be analyzed at once. Quantitative PCR was performed on a 7000 Fast Real-Time PCR System (Applied Biosystems, Foster City, CA, USA) using FastSYBR mixture (CWBIO) and group-specific bacterial 16S rDNA gene primers (Barman et al., 2008) (Supplementary Table 1). The results are presented as the abundance of each bacteria relative to total bacteria (eubacteria).

### Real-Time PCR for Colons and IECs

The colons were immediately frozen in liquid nitrogen and homogenized with Trizol (Life Technologies, Carlsbad, CA, USA). Total RNA was isolated from the homogenized colons according to the manufacturer's instructions for Trizol. cDNA was synthesized from 1 μg total RNA using a RevertAid RT Reverse Transcription Kit (Thermo Fisher Scientific, Waltham, MA, USA). Quantitative real-time PCR was performed using FastSYBR mixture (CWBIO) with specific primers (Supplementary Table 1). An internal control (*Actb*) was used to normalize the expression of target genes. For the IEC analysis, before Trizol treatment, the IECs were isolated as previously described (Becker et al., 2004) with minor modifications. Briefly, the colons were cut longitudinally and washed twice with cold PBS to remove fecal material. Subsequently, 5-mm-long pieces of colon were incubated at 37°C in PBS supplemented with 0.145 mg/ml dithiothreitol (DTT) and 0.37 mg/ml ethylenediaminetetraacetic acid (EDTA) for 15 min followed by vigorous stirring. The supernatant was passed through a 70-μm cell strainer and centrifuged at 1,000 rpm for 5 min, the pelleted IECs were collected. Total RNA was isolated from the pelleted IECs according to the manufacturer's instructions for Trizol. cDNA was synthesized as mentioned above. The cDNA was then subjected to further analysis by Quantitative real-time PCR.

### 16S rDNA Analysis of Fecal Samples

After extracting fecal DNA as mentioned above, the sequencing was carried out on an Illumina MiSeq platform (Illumina, San Diego, CA, USA) by OE Biotech Company (Shanghai, China). Each fecal DNA sample was used as a template for PCR amplification of the bacterial V3–V4 variable regions of 16S rDNA genes with specific primers containing a barcode. Trimmomatic software was used to detect and remove ambiguous bases and low-quality sequences. FLASH software was used to assemble paired-end reads. QIIME software was used for denoising and removing chimera. Vsearch software was used to generate operational taxonomic units (OTUs); sequences with >97% similarity were assigned to the same OTUs. Each representative read was annotated and blasted using Silva database version 123 (or Greengens) with RDP classifier (confidence threshold was 70%).

### Statistical Analyses

The statistical analyses were performed using Prism 8.0 (GraphPad Software, San Diego, CA, USA). Microbiome relative abundance analysis between two groups was evaluated using the two-tailed Mann–Whitney *U* test. The log-rank test was used to compare the mouse survival rate between two groups. Other comparisons between pairs of groups were analyzed using two-tailed Student's *t*-test. The data are presented as mean ± SEM. *P*-value < 0.05 was considered statistically significant.

## Results

### FAM96A Prevents Intestinal Microbiota Dysbiosis

**Figure 1:**
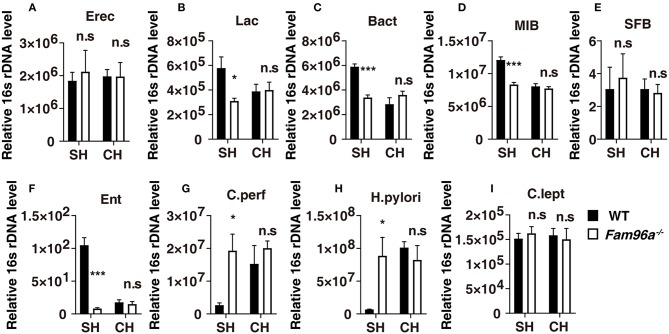
*Fam96a*^−/−^ mice harbor altered and transferable gut microbiota communities. **(A–I)** Real-time PCR analysis of relative 16S rDNA of several major bacterial groups in WT and *Fam96a*^−/−^ feces before and after cohousing for 8 weeks. Erec, *Eubacterium rectale/Clostridium coccoides*; Lac, *Lactobacillus* sp.; Bact, *Bacterioides* sp.; MIB, mouse intestinal *Bacterioides*; SFB, *Segmented Filamentous Bacteria*; Ent, *Enterobacteriaceae*; *C. perf*, *Clostridium perfringens*; *H. pylori, Helicobacter pylori*; *C. lept, Clostridium leptum*. SH, single housed; CH, cohoused; Data are expressed as mean ± SEM. *P < 0.05; ***P < 0.001. n.s., not significant. *n* = 6. Data are representative of three independent experiments.

We generated *Fam96a*^−/−^ mice and the deletion efficiency was validated by PCR and western blotting (Supplementary Figure 1). We first tested the impact of *Fam96a* deficiency on the composition of the total commensal bacteria. Several of the most common intestinal microbiota bacterial groups were analyzed by real-time PCR (Figure 1) (Barman et al., 2008). Compared with their littermate WT mice, a significant shift of colonic microflora composition was observed in the feces of *Fam96a*^−/−^ mice, with increases in *Clostridium perfringens* and *Helicobacter pylori* (Figures 1G,H), and notable decreases in *Lactobacillus, Bacteroides*, mouse intestinal *Bacteroides* and *Enterobacteriaceae* (Figures 1B,C,D,F). However, the levels of *Eubacterium rectale/Clostridium coccoides, Segmented Filamentous Bacteria, Clostridium leptum* (Figures 1A,E,I) seemed unchanged.

We then tested the fecal microbiota of cohoused WT and *Fam96a*^−/−^ mice by real-time PCR. Via coprophagia among mice, gut bacteria can be transferred between hosts by cohousing. Interestingly, after cohousing, WT and *Fam96a*^−/−^ mice exhibited a similar gut microbial composition, which was like that of single-housed *Fam96a*^−/−^ mice. Together, these data indicate that *Fam96a* depletion results in a distinct colonic microbiota configuration, and the microbiota from *Fam96a*^−/−^ mice can colonize WT mice, resulting in microbial dysbiosis.

### FAM96A Regulates AMP Expression in a Microbiota-Dependent Manner

**Figure 2:**
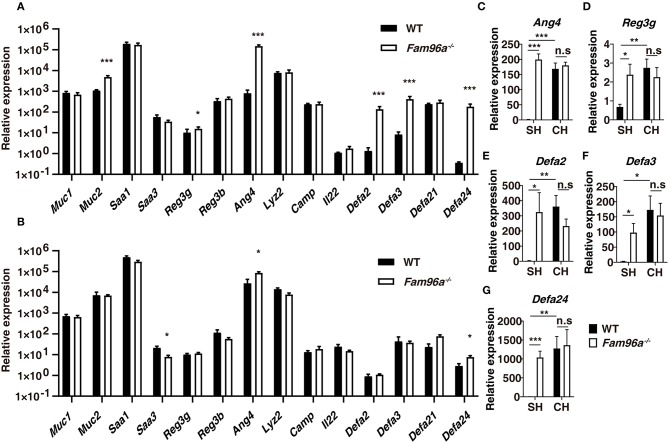
FAM96A regulates colonic antimicrobial peptide (AMP) genes in a microbiota-dependent manner. **(A)** mRNA levels of several AMPs in the colons of WT and *Fam96a*^−/−^ mice. **(B)** Mice were treated with a mixture of antibiotics for 2 weeks to deplete the intestinal microbiota, and the mRNA level in WT and *Fam96a*^−/−^ colons were then analyzed. **(C,D)** mRNA levels of indicated AMPs (*Ang4, Reg3g, Defa2, Defa3, Defa24*) in WT and *Fam96a*^−/−^ colons before **(C)** and after **(D)** cohousing. Data are expressed as mean ± SEM. *P < 0.05; **P < 0.005; ***P < 0.001. For **(A,B)**, *n* = 6. For **(C–G)**, *n* = 5. Data are representative of three independent experiments.

Intestinal epithelial AMPs have an essential role in maintaining gut microbiota homeostasis and allowing epithelial surfaces to manage the surrounding microorganisms. On the other hand, the microbiota mutually regulates the expression and secretion of many intestinal AMPs (Gallo and Hooper, 2012). To determine whether FAM96A prevents gut microbial dysbiosis via AMPs, we tested the AMP expression by performing real-time PCR using colonic tissue from *Fam96a*^−/−^ and WT mice. As shown in Figure 2A, the transcription levels of several AMPs were elevated in *Fam96a*^−/−^ mice, including *Defa2, Defa3, Defa24, Ang4*, and *Reg3g*. In addition, the mRNA level of *Muc2*, a mucin mainly produced by goblet cells, was also increased in *Fam96a*^−/−^ colons (Figure 2A). We next assessed whether the effect of *Fam96a* on AMPs was microbiota dependent by treating *Fam96a*^−/−^ and WT mice with antibiotics for 2 weeks to deplete the intestinal microbiota. After the antibiotic treatment, the AMP expression in both *Fam96a*^−/−^ and WT colons decreased to a similar level (Figure 2B); the fold change of *Ang4* reduced from 186 to 2.3 and the fold change of *Defa24* decreased from 102 to 2.7 (Figures 2A,B). This indicates a potential role of the gut microbiota in regulating AMPs in *Fam96a*^−/−^ colons. To further assess the regulatory role of the gut microbiota, we cohoused WT and their littermate *Fam96a*^−/−^ mice for 8 weeks, and the previously altered AMPs were then retested. As shown in Figures 2C–G, all the AMPs tested exhibited no differences between the cohoused WT and *Fam96a*^−/−^ mice. Taken together, these findings suggest that FAM96A regulates the AMP expression by controlling the microbiota ecosystem.

### FAM96A Maintains Colonic Crypt Homeostasis and Goblet Cell Numbers by Influencing the Gut Microbiota Composition

**Figure 3:**
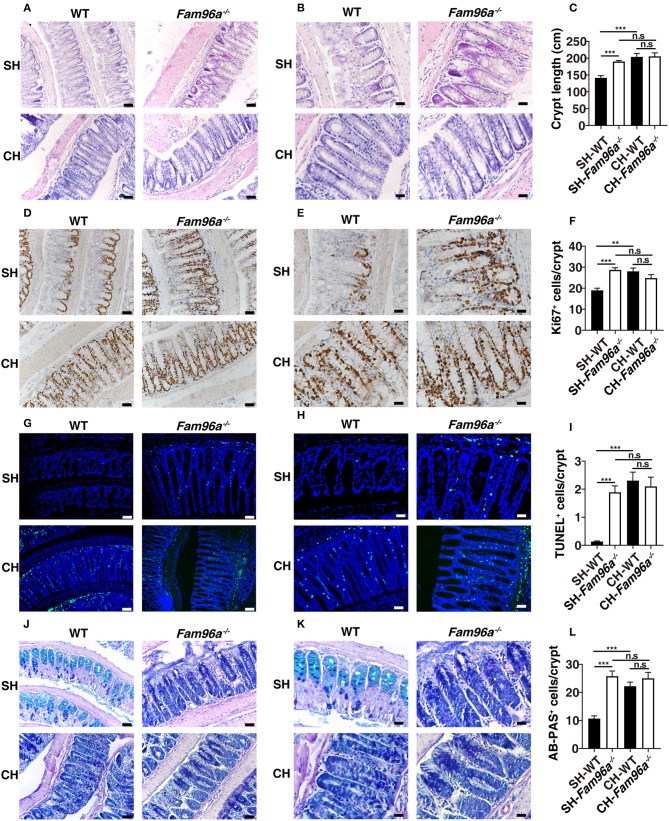
The altered colonic epithelial phenotype is affected by housing conditions. WT and *Fam96a*^−/−^ mice were cohoused for 8 weeks, and the colons from the cohoused mice and the gender-/age-matched single-housed mice were then collected and analyzed. **(A,B)** Representative HE staining of sections of WT and *Fam96a*^−/−^ colons. **(C)** Quantification of crypt height. **(D,E)** Representative Ki67 immunohistochemistry staining. **(F)** Quantification of proliferative colonic epithelial cells (Ki67^+^ cells/crypts). **(G,H)** Representative TUNEL staining. **(I)** Quantification of apoptotic colonic epithelial cells (TUNEL^+^ cells/crypts). **(J,K)** Representative Alcian blue/periodic acid–Schiff (AB-PAS) staining of goblet cells. **(L)** AB-PAS-positive cells in each crypt were enumerated. Scale bars, for **(A,D,G,J)**, 50 μm, for **(B,E,H,K)**, 25 μm. Data are expressed as mean ± SEM. *P < 0.05; **P < 0.005; ***P < 0.001. n.s., not significant. *n* = 4–5. Data are representative of three independent experiments.

We then assessed whether FAM96A influences colonic IECs *in vivo*. HE staining was performed to histologically characterize colons from *Fam96a*^−/−^ mice and littermate WT mice. Morphometric analysis demonstrated that *Fam96a*^−/−^ mice had greater crypt height (Figures 3A–C). TUNEL assays and Ki67 staining were performed and the results showed enhanced apoptosis and proliferation of *Fam96a*^−/−^ colonic IECs (Figures 3D–I). These data indicate the imbalance of colonic IEC turnover in *Fam96a*^−/−^ mice, which may be the cause of the thickened mucosa. Goblet cells, which are interspersed among enterocytes, are very important in colonic homeostasis. They secrete gel-forming mucins and make up the skeletons of the gut mucus layer (Johansson and Hansson, 2016). Consistent with the increased *Muc2* mRNA level in colons (Figure 2A), PAS staining revealed an increased number of goblet cells in the colon of *Fam96a*^−/−^ mice (Figures 3J–L). Together, these findings indicate that FAM96A can regulate the intestinal epithelial phenotype, including crypt morphology, IEC proliferation and apoptosis, and goblet cell numbers. Commensal bacteria are involved in the regulation of IEC turnover and goblet cell development (Rakoff-Nahoum et al., 2015; Yu et al., 2016). To verify whether the altered intestinal epithelial phenotype was related to the microbiota, we cohoused WT mice with *Fam96a*^−/−^ mice. After 8 weeks of cohousing, the colonic epithelial differences were abrogated and all mice had a phenotype similar to single-housed *Fam96a*^−/−^ mice. As shown in Figure 3, the colonic crypt length of cohoused WT mice increased to the level in *Fam96a*^−/−^ mice, with increased proliferative and apoptotic IECs (Figures 3A–F). In addition, AB-PAS staining indicated that the goblet cell numbers in the WT mice were also increased to the *Fam96a*^−/−^ mice level (Figures 3G,H). These results might be explained by the fact that the gut microbial profiles of WT mice were transformed to a *Fam96a*^−/−^ profile after cohousing. Thus, our data suggest that the disturbed intestinal homeostasis in *Fam96a*^−/−^ mice, which is transferable, is induced by microbial dysbiosis.

### FAM96A Regulates Intestinal Barrier Permeability by Influencing the Colonic Microbiota

**Figure 4:**
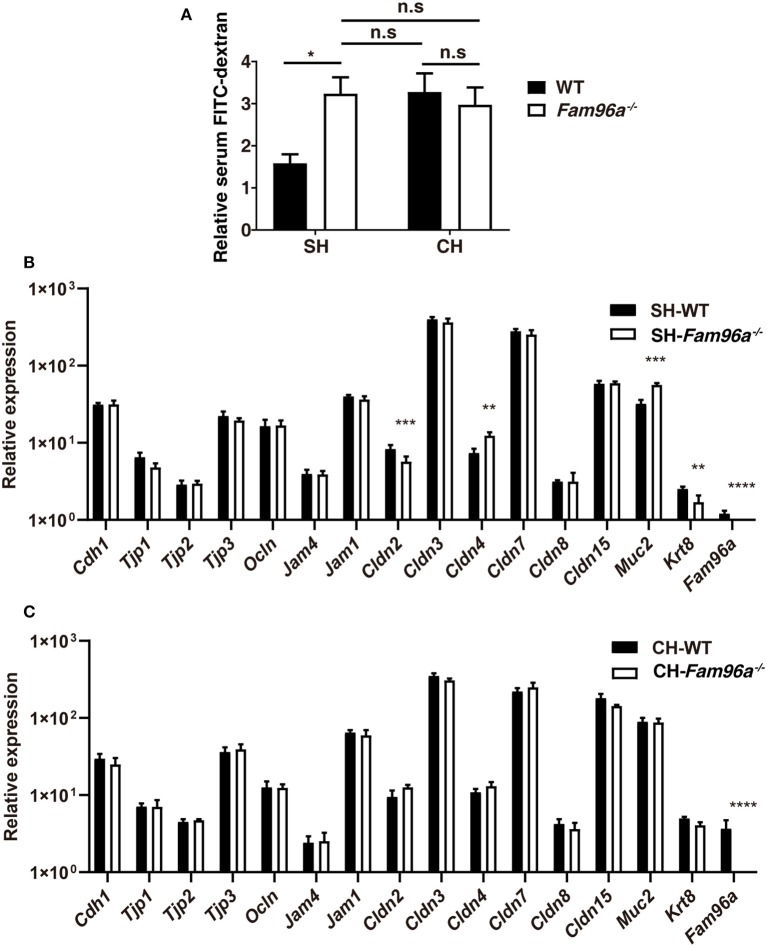
FAM96A plays a role in protection against epithelial barrier permeabilization. **(A)** Relative concentration of FITC-dextran in WT and *Fam96a*^−/−^ mice serum before and after cohousing for 8 weeks. **(B,C)** Real-time PCR analysis of TJ proteins in IECs from single-housed **(B)** and cohoused **(C)** mice. SH, single housed; CH, cohoused. Data are expressed as mean ± SEM. *P < 0.05; **P < 0.005; ***P < 0.001; ****P < 0.0001. For **(A)**, *n* = 4–5. For **(B,C)**, *n* = 5–6. Data are representative of three independent experiments.

Alteration in IEC turnover is a potential contributor to gut barrier dysfunction (Günther et al., 2014). To assess the impact of FAM96A on the function of the gut epithelial barrier, we assessed the intestinal permeability of WT and *Fam96a*^−/−^ mice. Mice were gavaged with 4 kDa FITC-dextran and, 4 h later, the concentration of serum FITC-dextran was measured as an indicator of intestinal permeability. At baseline, the intestinal permeability of *Fam96a*^−/−^ mice was higher than that of WT mice, indicating epithelial barrier dysfunction upon *Fam96a* depletion (Figure 4A). During the development and migration of IECs, epithelial TJs are formed at the cell–cell contact points to seal off gaps between cells (Zihni et al., 2016). TJ complexes are key players in maintaining the epithelial barrier, preventing bacteria influx via paracellular routes (Zihni et al., 2016). Real-time PCR results showed that the mRNA level of several TJ related proteins, including Claudin-2, Claudin-4, and Krt-8, were altered in *Fam96a*^−/−^ IECs (Figure 4B), indicating a regulatory role of FAM96A in TJs. The commensal microbiota also participate in epithelial reconstitution and reorganization of TJs (Zareie et al., 2006; Miyauchi et al., 2009), so we next assessed, by cohousing, whether the microbiota also plays a role in regulating the intestinal permeability. As expected, cohoused *Fam96a*^−/−^ mice had a comparable intestinal permeability to WT mice (Figure 4A), which matched the alterations in the epithelial phenotype upon cohousing. Notably, the expression of TJ proteins in the two cohoused groups was also similar (Figure 4C). Collectively, these findings suggest that FAM96A regulates the expression of TJ proteins and gut permeability by influencing the gut microbiota composition.

### *Fam96a^−/−^* Mice Are More Susceptible to DSS-Induced Acute Colitis

**Figure 5:**
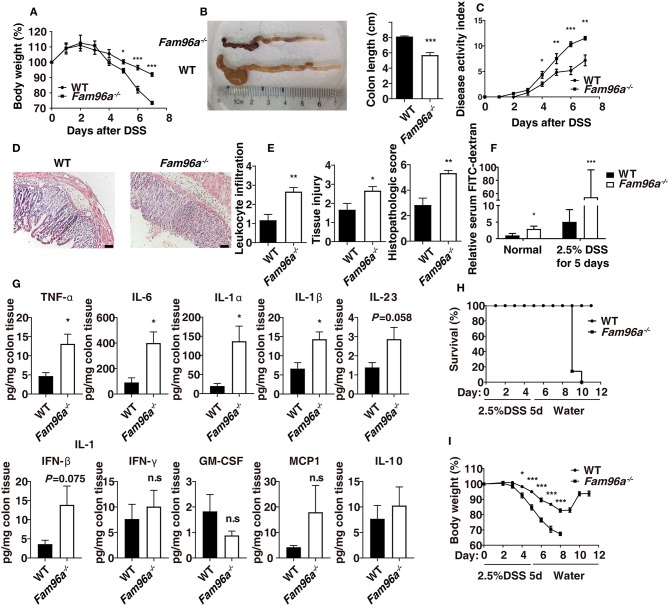
FAM96A protects mice from DSS-induced colitis. **(A)** Body weights of the two groups of mice. **(B)** Colon length (at day 7 post DSS treatment). **(C)** DAI (assessed every day). **(D)** HE staining of colon sections and **(E)** Histopathologic scores based on a double-blind method. **(F)** DSS-treated mice and gender-/age-matched controls were gavaged with FITC-dextran, and the FITC-dextran concentration in the serum was then determined 4 h later. Relative concentrations are shown. **(G)** Cytokine expression was assessed using a LegendPlex Mouse Inflammation Panel kit. Mice were given 2.5% DSS for 5 days followed by normal drinking water; survival rate **(H)** and body weight **(I)** were monitored every day. Scale bars, 50 μm. Data are expressed as mean ± SEM. *P < 0.05; **P < 0.005; ***P < 0.001. *n* = 6–7. For **(A–E)**, data are representative of five independent experiments. For **(F–H)**, data are representative of three independent experiments.

Increasing evidence suggests an important role for gut microbiota in shaping inflammatory environments (Ivanov and Honda, 2012). In addition, compromised intestinal barrier function is considered to be a main factor in the pathogenesis of IBD (Salim and Söderholm, 2011). Given the gut microbial dysbiosis and the defects in intestinal barrier observed in *Fam96a*^−/−^ mice, we assessed whether *Fam96a* KO would change the colonic inflammation status. We treated *Fam96a*^−/−^ mice and their littermate WT mice with 3.0% DSS in the drinking water to induce colitis. *Fam96a*^−/−^ mice showed higher susceptibility to DSS compared with WT mice, as during colitis, they showed much greater body weight loss (Figure 5A), shorter colons (Figure 5B), and higher DAI (Figure 5C). HE staining revealed increased pathology in *Fam96a*^−/−^ colons, including immune cell infiltration, epithelial injury, crypt hyperplasia, and edema (Figures 5D,E). Additionally, at day 5 after DSS treatment, the intestinal permeability of *Fam96a*^−/−^ mice was greater than that of their WT counterparts (Figure 5F), which was in accordance with their higher levels of inflammation and poorer epithelial integrity. Severity of inflammation can be assessed by inflammatory cytokines. We collected the colons from WT and *Fam96a*^−/−^ mice at day 7 after DSS treatment, and a LegendPlex Inflammatory cytokines kit was used to detect the cytokines in the colon homogenate. As shown in Figure 5G, tumor necrosis factor (TNF)-α, interleukin (IL)-6, IL-1β, and IL-1α were elevated in the colons from *Fam96a*^−/−^ mice compared to WT mice. The expression of the anti-inflammatory cytokine IL-10 was similar between the two groups, suggesting that *Fam96a*'s protective role in colitis may not be IL-10 dependent.

To evaluate the repair process after acute mucosal injury, mice were provided 2.5% DSS in the drinking water for 5 days and then switched to normal drinking water for recovery, and body weight was recorded every day (Figure 5I). *Fam96a*^−/−^ mice could not recover after DSS challenge, and at day 8 post DSS administration, all the *Fam96a*^−/−^ mice died; on the other hand, all the WT mice eventually recovered from colitis and survived (Figure 5H). Collectively, these data indicate that FAM96A plays a protective role in DSS-induced colitis.

### Microbial Dysbiosis in *Fam96a^−/−^* Mice Is Colitogenic and Transferable

**Figure 6:**
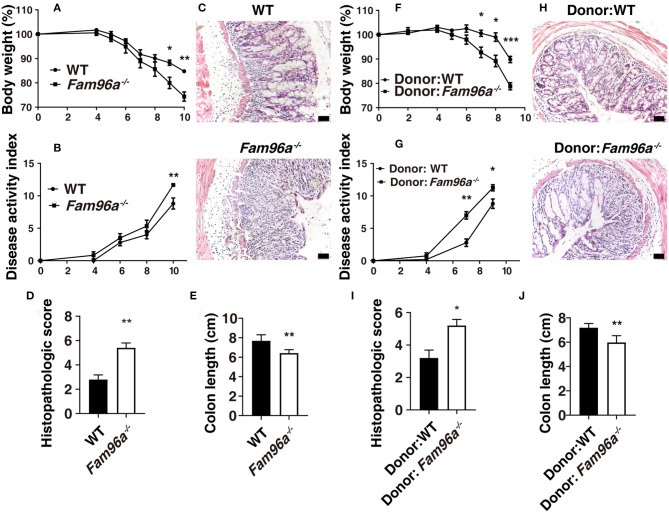
*Fam96a*^−/−^ mice have a transmissible and colitogenic gut microbiota. **(A–E)** WT and *Fam96a*^−/−^ mice were pretreated with a mixture of ampicillin, neomycin, metronidazole, and vancomycin for 2 weeks followed by 2.5% DSS treatment. The body weight **(A)**, DAI **(B)**, representative HE staining images **(C)**, histopathologic score **(D)**, and colon length **(E)** are shown. **(F–J)** WT mice were pretreated with the antibiotics mixture and then received either WT or *Fam96a*^−/−^ feces. After 2 weeks of FMT, the two groups of mice were challenged with 2.5% DSS. Colitis severity is indicated by body weight **(F)**, DAI **(G)**, histopathologic score **(I)**, HE staining **(H)**, and colon length **(J)**. Scale bars, 50 μm. Data are expressed as mean ± SEM. *P < 0.05; **P < 0.005; ***P < 0.001. For **(A–E)**, *n* = 5–6. For **(F–J)**, *n* = 4–5. Data are representative of three independent experiments.

The intestinal microbiota strongly affects the outcome and disease progress of colitis (Ivanov and Honda, 2012). To assess the role of gut microbiota in colitis in WT and *Fam96a*^−/−^ mice, we treated the mice with broad-spectrum antibiotics in drinking water for 2 weeks followed by inducing colitis with 2.5% DSS. It turns out that the *Fam96a*^−/−^ mice still had an increased colitis severity compared to WT mice after antibiotic treatment, with faster body weight loss (Figure 6A), higher DAI (Figure 6B), higher histopathologic score based on HE staining (Figures 6C,D), and shorter colons (Figure 6E). This suggested two possibilities: FAM96A might have an intrinsic influence on colonic cells or the antibiotics might be insufficient to eradicate all the microbes. To avoid interference from non-microbiota factors, we conducted FMT experiments. Feces from *Fam96a*^−/−^ mice and WT littermates were transferred into two groups of antibiotic-pretreated WT mice, respectively. FMT led to notable alterations in the microbial composition in the two recipient groups (Supplementary Figure 2), confirming the successful implementation of FMT. After 2 weeks of FMT treatment, the *Fam96a*^−/−^ feces recipients exhibited greater sensitivity to DSS-induced colitis than WT feces recipients, based on more profound weight loss (Figure 6F), higher DAI (Figure 6G), higher histological score (Figure 6I), shorter colons (Figure 6J), and greater destructive tissue damage and inflammatory cell infiltration, based on HE staining (Figure 6H). Taken together, these findings indicate that the colitis sensitivity in *Fam96a*^−/−^mice is conferred by the gut microbial dysbiosis, and the colitogenic microbiota is transmissible.

### 16S rDNA Sequencing Reveals in Detail the Alteration of Intestinal Microbial Composition in *Fam96a^−/−^* Mice

We used 16S rDNA sequencing to specifically examine the alteration in gut microbiota from WT and *Fam96a*^−/−^ mice before and after 5 days of 2.5% DSS treatment.

**Figure 7:**
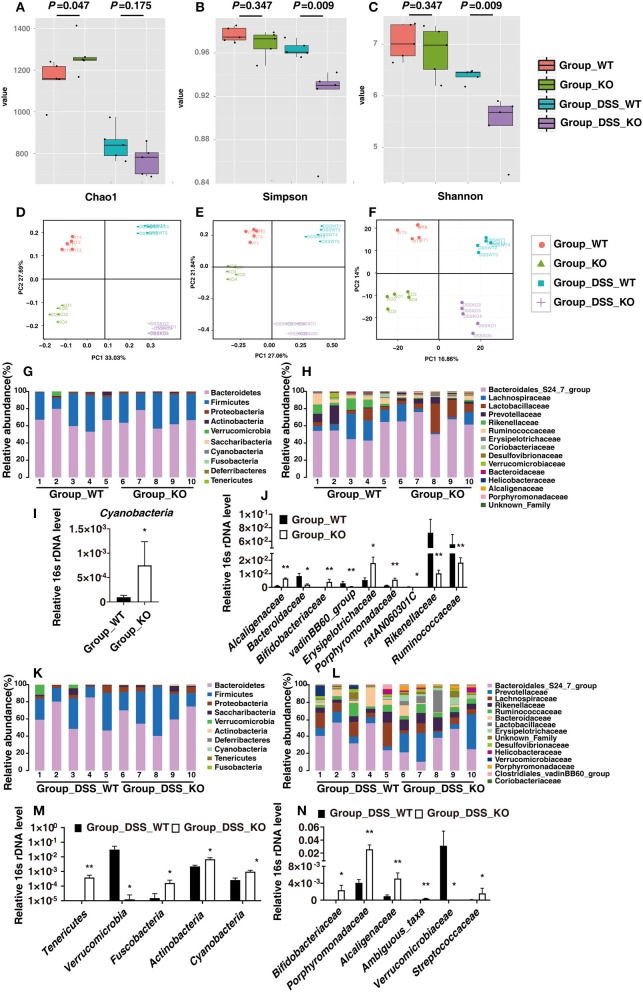
Relative gut bacteria levels in WT and *Fam96a*^−/−^ mice at baseline and at day 5 after 2.5% DSS treatment. **(A–F)** α- and β-diversity of the fecal microbiota in Group_WT, Group_KO, Group_DSS_WT, and Group_DSS_KO. **(A)** Chao 1. **(B)** Simpson index. **(C)** Shannon index. **(D)** Principal coordinate analysis (PCoA) of weighted UniFrac distances. **(E)** PCoA analysis of Bray-Curtis dissimilarity. **(F)** Principal component analysis (PCA) of gut bacteria. α-diversity data are expressed as mean ± SEM. *n* = 5. **(G–J)** Analysis of commensal bacteria at the phylum **(G,I)** and family **(H,J)** levels at baseline. **(I)** Relative abundance of the phylum *Cyanobacteria*. **(J)** Relative abundance of the families *Alcaligenaceae, Bacteroidaceae, Bifidobacteriaceae, Clostridiales_vadinBB60*_group, *Erysipelotrichaceae, Porphyromonadaceae, ratAN060301C, Rikenellaceae*, and *Ruminococcaceae*. **(K–N)** Quantification of bacterial phyla **(K,M)** and families **(L,N)** with respect to genotype (*Fam96a*^−/−^ and WT) during colitis. **(M)** Relative abundance of the phyla *Tenericutes, Verrucomicrobia, Fusobacteria, Actinobacteria*, and *Cyanobacteria* during colitis. **(N)** Relative abundance of the families *Bifidobacteriaceae, Porphyromonadaceae, Alcaligenaceae, Verrucomicrobiaceae*, and *Streptococcaceae* during colitis. Data are expressed as mean ± SEM. *P < 0.05; **P < 0.005 (Mann–Whitney *U* test). *n* = 5.

Chao1, the Shannon index, and the Simpson index are commonly used to evaluate the α-diversity of the microbiota. At baseline, there were no significant differences in the Simpson and Shannon indexes between the samples from WT and *Fam96a*^−/−^ mice (Figures 7B,C), though Chao1 was a little higher in Group_KO (Figure 7A). After DSS treatment, the Chao1 of both groups was dramatically reduced, while the Shannon and Simpson indexes of the *Fam96a*^−/−^ mice (Group_DSS_KO) were much lower than those of the WT mice (Group_DSS_WT) (Figures 7A–C). Of note, the *P*-values for the comparisons of the Shannon and Simpson indexes between Group_WT and Group_KO were the same as those for the comparisons between Group_DSS_WT and Group_DSS_KO, which was just a coincidence. These results reveal that DSS decreased the biodiversity of the intestinal microbiota, and the biodiversity changed more significantly in *Fam96a*^−/−^ mice during colitis. As for β-diversity, the unweighted UniFrac-principal coordinates analysis (PCoA) (Figure 7D), the PCoA plots of Bray–Curtis dissimilarity (Figure 7E), and the principal component analysis (PCA) (Figure 7F) showed that the microbiomes of each of the four groups clustered separately, confirming the distinct microbial profiles.

We next analyzed the bacteria with relatively high abundance whose composition showed statistically significant discrepancies between the WT and *Fam96a*^−/−^ mice. At baseline, relative to WT mice, *Fam96a*^−/−^ mice had an increased relative abundance of *Cyanobacteria* at the phylum level (Figures 7G,I). At the family level, *Fam96a*^−/−^ mice had increased relative abundance of *Alcaligenaceae, Bifidobacteriaceae, Erysipelotrichaceae*, and *Porphyromonadaceae* and reduced relative abundance of *Bacteroidaceae, Clostridiales_vadinBB60_*group, *ratAN060301C, Rikenellaceae*, and *Ruminococcaceae* (Figures 7H, J). During colitis, at the phylum level, the relative abundance of *Tenericutes, Fusobacteria, Actinobacteria*, and *Cyanobacteria* in Group_DSS_KO was increase, while the relative abundance of *Verrucomicrobia* was decreased (Figures 7K,M). At the family level, *Fam96a*^−/−^ mice had elevated relative abundance of *Bifidobacteriaceae, Porphyromonadaceae, Alcaligenaceae, Streptococcaceae*, and *Ambiguous_taxa* and a reduced relative abundance of *Verrucomicrobiaceae* (Figures 7L,N). These results further illustrate the detailed differences between gut microbial communities in *Fam96a*^−/−^ and WT mice.

## Discussion

The commensal microbiota is a key part of the intestinal barrier system, and microbial dysbiosis often leads to increased pathological responses in the gut (Thomas, 2018). Real-time PCR revealed increased levels of inflammation-promoting bacteria in *Fam96a*^−/−^ feces, such as *Helicobacter* and *Clostridium perfringens*, and a decreased level of the probiotic *Lactobacillus/Lactococcus*. This indicates that *Fam96a*^−/−^ mice may harbor pro-inflammatory gut microbiota profiles. 16S rDNA sequencing indicated an overall microbial dysbiosis in the *Fam96a*^−/−^ gut.

Intestinal epithelial AMPs directly kill or inhibit the growth of microorganisms to maintain microbial homeostasis. In contrast, the expression of many intestinal AMPs can be regulated by the microbiota (Gallo and Hooper, 2012). Dramatically increased levels of some AMPs, including *Ang4, Reg3*γ, and several α-defensins in *Fam96a*^−/−^ mice colons were observed. After antibiotic treatment, the increased expression was partially abrogated. In addition, after 8 weeks of cohousing, the AMPs in the colons of WT and *Fam96a*^−/−^ mice exhibited similar expression levels, thus suggesting that FAM96A regulates the AMP expression in a microbiota-dependent manner. *In vivo* research has revealed that *Reg3*γ and α-defensins play a protective role against pathogen invasion and colonization in mice intestines (Gallo and Hooper, 2012). Downregulation of *Ang4* by overexpression of tumor necrosis factor alpha-induced protein 3 (TNFAIP3) in IECs is associated with luminal microbe invasion (Murphy et al., 2014). Transgenic mice expressing human *DEFA5* had greater resistance to oral challenge with *S*. Typhimurium than WT mice (Salzman et al., 2003). Thus, these studies indicate that AMPs usually seem to play a protective role in the gut. Given these studies and the profound increase in opportunistic pathogens observed in the *Fam96a*^−/−^ colon, we speculate that the enhanced expression of AMPs in the *Fam96a*^−/−^ colon may be a consequence, but not the cause, of microbial dysbiosis. However, we could not rule out the possibility that some unknown AMPs may be involved in the regulation of the microbial ecology in *Fam96a*^−/−^ mice.

Commensal bacteria are involved in the regulation of IEC turnover, promotion of epithelial restitution, development of goblet cells (Yu, 2012), and reorganization of TJs, all of which are pivotal for ensuring strong barrier function. Disruption to the gut microbial ecology may cause disordered intestinal epithelium homeostasis (Yu, 2012). In accordance with the microbial dysbiosis, there were indeed some subtle alterations in *Fam96a*^−/−^ colonic epithelial cells such as enhanced crypt height and dysregulated cell turnover. Goblet cells are specialized IECs distributed throughout the intestinal tract that secrete gel-forming mucins, providing the first line of defense in the host gut (Johansson and Hansson, 2016). Goblet cell hyperplasia was observed in the colons of *Fam96a*^−/−^ mice on AB-PAS staining. The intestinal barrier function was disrupted in *Fam96a*^−/−^ mice, as we observed an increase in *Fam96a*^−/−^ intestinal barrier permeability. Intestinal permeability is mainly regulated by gut epithelial TJs, which are composed of transmembrane proteins, including various claudins (CLDNs), TJ-associated MARVEL domain-containing proteins (TAMPs, such as occludin and junctional adhesion molecules), and cytosolic proteins that connect transmembrane components to the cytoskeleton (Zeisel et al., 2018). TJ breakdown allows material from the lumen to penetrate the adluminal compartment of the epithelium and causes inflammation. TJ alteration is implicated in a variety of gastrointestinal diseases including IBD (Zeisel et al., 2018). Many pathogenic bacteria target specific TJ proteins, leading to various diseases (Zihni et al., 2016). In accordance with this, we found abnormal mRNA expression of several TJ-related proteins in *Fam96a*^−/−^ mice colons, including upregulation of Claudin-4 and downregulation of Claudin-2 and Keratin-8. This dysregulation of TJ-related proteins may underlie the intestinal barrier defects in *Fam96a*^−/−^ intestines. After cohousing *Fam96a*^−/−^ mice with WT mice, which allowed full microbiota transfer between the two groups, the mice of both genotypes exhibited a similar colonic epithelial phenotype to that observed in single-housed *Fam96a*^−/−^ mice. Additionally, the differences in gut permeability and TJ-related protein expression between the two groups of mice were also abrogated. This was consistent with the fact that the cohoused WT mice showed a dramatic shift in their gut bacterial composition toward the composition in single-housed *Fam96a*^−/−^ mice, while the microbiota in cohoused *Fam96a*^−/−^ mice seemed unaffected. These findings indicate that *Fam96a* depletion-induced changes in the microbiota are transmissible to WT mice and are responsible for the altered intestinal epithelial phenotypes in cohoused WT mice, indicating causality.

DSS-induced colitis is a widely used animal model for investigating the pathogenesis of IBD. DSS damages the IECs, allowing direct contact of gut microbes with host cells, resulting in intestinal inflammation (Perše and Cerar, 2012). FAM96A appears to protect mice from colitis, as *Fam96a*^−/−^ mice developed more severe DSS-induced colitis than WT mice. DSS treatment after microbiota depletion by antibiotics showed that *Fam96a*^−/−^ mice were still more hypersensitive to colitis than WT mice, which may be caused by some intrinsic alteration in host cells upon *Fam96a* deletion, or by the incomplete clearance of gut microbes. In the DSS experiment following FMT treatment, WT mice that received *Fam96a*^−/−^ feces were more susceptible to DSS than those that received microbiota from WT mice, confirming the colitogenic and transferable characteristics of the *Fam96a*^−/−^ intestinal microbiota.

This study investigated the role of *Fam96a* in colonic homeostasis and in colon injury. The deletion of *Fam96a* in mice resulted in a dramatic shift in the gut microbial community, altered the colonic epithelial cell phenotype, compromised the intestinal barrier, and exacerbated the DSS-induced colitis. The alteration of the colon phenotype and barrier function and enhanced susceptibility to colonic inflammation observed in *Fam96a*^−/−^ mice was transferable to WT mice via feces, demonstrating that the regulatory role of *Fam96a* is largely dependent on the intestinal microbiota. Our study reveals that FAM96A plays a protective role in DSS-induced colitis by maintaining the gut microbiota ecology, provides information on the *in vivo* function of *Fam96a* for the first time, and provides novel evidence regarding host–microbe interactions.

## Data Availability Statement

The 16S rDNA sequencing data has been deposited in NCBI repository, SRA number is PRJNA547859.

## Ethics Statement

This study was carried out in accordance with the recommendations of the guidelines approved by the Institutional Animal Care and Use Committee of Peking University. The protocol was approved by the Institutional Animal Care and Use Committee of Peking University.

## Author Contributions

AY contributed to the design of the study, performing the experiments, data analysis and interpretation, and writing the manuscript. YL contributed to the design of the study, performing the experiments, and data analysis. WC and MH assisted with some animal experiments. JD, NZ, and LC prepared the experimental reagents. LW contributed essential ideas and discussion, and supervised the work.

### Conflict of Interest

The authors declare that the research was conducted in the absence of any commercial or financial relationships that could be construed as a potential conflict of interest.
