# Supplementary material for: FAM96A Protects Mice From Dextran Sulfate Sodium (DSS)-Induced Colitis by Preventing Microbial Dysbiosis
Source: Front Cell Infect Microbiol. 2019 Nov 18;9:381. doi: 10.3389/fcimb.2019.00381 (PMC6876263; doi:10.3389/fcimb.2019.00381)
Supplement: Supplementary file 1 [file Table_1.DOCX]

***Supplementary Materials***

**
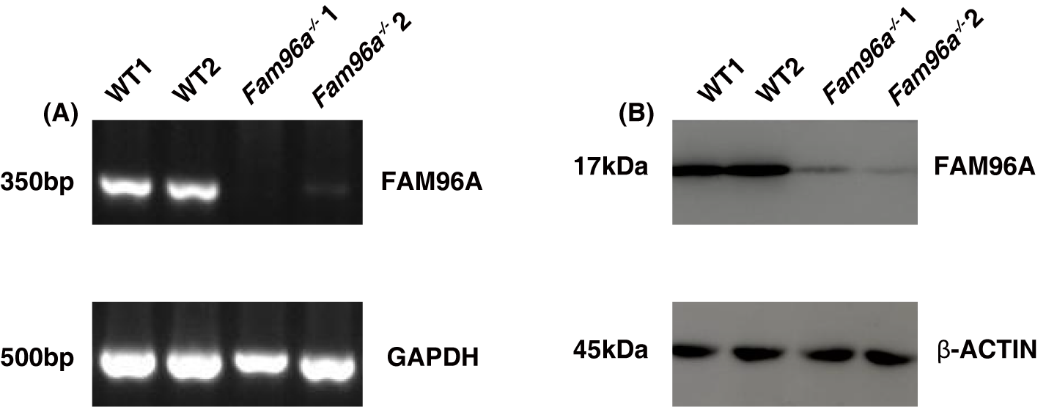
 Supplementary Figure 1.** Analysis of FAM96A expression in colons from WT and *Fam96a^-/-^* mice. Two WT mice and two *Fam96a^-/-^* mice were used to detect the efficiency of *Fam96a* deletion. **(A)** Colonic RNA was isolated and reversed transcribed to cDNA, and PCR was performed to detect the mRNA expression of *Fam96a* relative to *Gapdh*. **(B)** Western blotting was used to detect the protein expression of FAM96A.


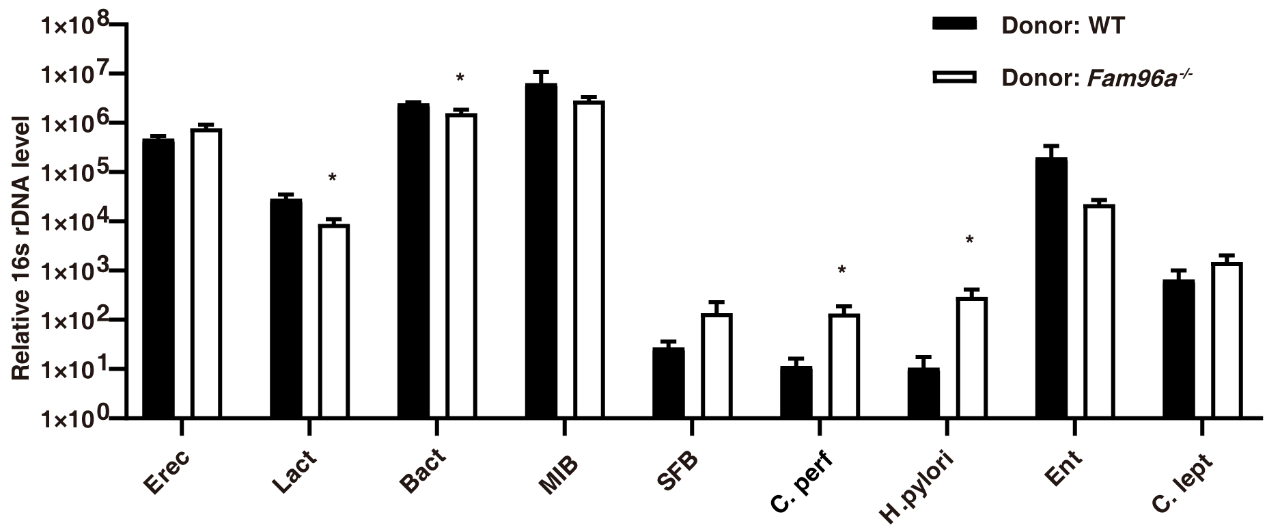


**Supplementary Figure 2.** Analysis of bacterial 16S rDNA by real-time PCR in the two groups of WT mice after receiving FMT. Erec, *Eubacterium rectale/Clostridium coccoides*; Lac, *Lactobacillus* sp.; Bact, *Bacterioides* sp.; MIB, mouse intestinal *Bacterioides*; SFB, *Segmented Filamentous Bacteria*; Ent, *Enterobacteriaceae*; C.perf, *Clostridium perfringens;* H.pylori, *Helicobacter pylori*; C.lept, *Clostridium leptum*. SH, single housed; CH, cohoused. Data are expressed as mean ± SEM. **P*<0.05. n=6. Data are representative of three independent experiments.

**Supplementary Table 1**: Primers used in the study

Bacterial 16s rDNA gene primers.

| Bacteria | Primer sequence |
| --- | --- |
| *Eubacteria（all bacteria）* | F：ACTCCTACGGGAGGCAGCAGT |
|  | R：ATTACCGCGGCTGCTGGC |
| *Eubacterium rectale* | F：ACTCCTACGGGAGGCAGC |
|  | R：GCTTCTTAGTCAGGTACCGTCAT |
| *Lactobacillus/Lactococcus* | F：AGCAGTAGGGAATCTTCCA |
|  | R：CACCGCTACACATGGAG |
| *Bacteroides* | F：GGTTCTGAGAGGAGGTCCC |
|  | R：GCTGCCTCCCGTAGGAGT |
| *Mouse intestinal Bacteroides* | F：CCAGCAGCCGCGGTAATA |
|  | R：CGCATTCCGCATACTTCTC |
| *Segmented filamentous bacteria* | F：GACGCTGAGGCATGAGAGCAT |
|  | R：GACGGCACGGATTGTTATTCA |
| *Clostridium perfringens* | F：CGCATAACGTTGAAAGATGG |
|  | R：CCTTGGTAGGCCGTTACCC |
| *Helicobacter* | F：TTAACCATAGAACTGCATTTGAAACTAC |
|  | R：GGTCGCCTTCGCAATGAGTA |
| *Clostridium leptum* | F：GCACAAGCAGTGGAGT |
|  | R：CTTCCTCCGTTTTGTCAA |
| *Enterobacteriaceae* | F: GTGCCAGCMGCCGCGGTAA |
|  | R :GTGCCAGCMGCCGCGGTAA |

Primers used in the Real-time PCR for colons or intestinal epithelial cells.

| Gene name | Primer sequence |
| --- | --- |
| *Fam96a* | F: GAACAAAGGAGCACCACAAA |
|  | R: AGACCTGCTAAGTCATCCCG |
| *Cdh1* | F：ACCAGCAGTTCGTTGTTGTCAC |
|  | R：GTTCCTCGTTCTCCACTCTCA |
| *Ocln* | F：ACGGACCCTGACCACTATGA |
|  | R：TCAGCAGCAGCCATGTACTC |
| *Muc2* | F：GCTGACGAGTGGTTGGTGAATG |
|  | R：GATGAGGTGGCAGACAGGAGAC |
| *Muc1* | F：GCAGTCCTCAGTGGCACCTC |
|  | R：CACCGTGGGCTACTGGAGAG |
| *Muc3* | F：CGTGGTCAACTGCGAGAATGG |
|  | R：CGGCTCTATCTCTACGCTCTCC |
| *Camp* | F：CTTCAACCAGCAGTCCCTAGACA |
|  | R：TCCAGGTCCAGGAGACGGTA |
| *Cldn2* | F：GGCTGTTAGGCACATCCAT |
|  | R：TGGCACCAACATAGGAACTC |
| *Cldn3* | F：AAGCCGAATGGACAAAGAA |
|  | R：CTGGCAAGTAGCTGCAGTG |
| *Cldn4* | F：CGCTACTCTTGCCATTACG |
|  | R：ACTCAGCACACCATGACTTG |
| *Cldn7* | F：AGGGTCTGCTCTGGTCCTT |
|  | R：GTACGCAGCTTTGCTTTCA |
| *Cldn8* | F：GCCGGAATCATCTTCTTCAT |
|  | R：CATCCACCAGTGGGTTGTAG |
| *Cldn15* | F：CAGCTTCGGTAAATATGgCA |
|  | R：CAGTGGGACAAGAAATGGTG |
| *Lys2* | F：GTGAGAGATCCCCAAGGCAT |
|  | R：GAGGGGAAATCGAGGGAATG |
| *Jam1* | F：ACCCTCCCTCCTTTCCTTAC |
|  | R：CTAGGACTCTTGCCCAATCC |
| *Jam4* | F：GGACTCAGAGGCTCACTTCA |
|  | R：AGACTCAGCACCACCATTTG |

| *Tjp1（ZO1）* | F：AGGACACCAAAGCATGTGAG |
| --- | --- |
|  | R：GGCATTCCTGCTGGTTACA |
| *Tjp2（ZO2）* | F：ATGGGAGCAGTACACCGTGA |
|  | R：TGACCACCCTGTCATTTTCTTG |
| *Tjp3（ZO3）* | F：TCGGCATAGCTGTCTCTGGA |
|  | R：GTTGGCTGTTTTGGTGCAGG |
| *Krt8* | F：AAGGTGTGGAAAGAGCTAGCC |
|  | R：ATAGACGTTGACTGCACGCACT |
| *Defa2* | F：GGCTCCTGCTCACCAATTCT |
|  | R：GATCAGCCTGGACCTGGAAG |
| *Defa3* | F：TCGCTGAACATGGAGACCAC |
|  | R：CGAGGTAGTCATCAGGCACC |
| *Defa21* | F：CGCTGAGAGTGCAGATGACA |
|  | R：GAAGTGTTCATCAGGCCCCA |
| *Defa24* | F：ACACTGAGCTGCTACTCACC |
|  | R：AGACACAGCCTGGTCCTCTT |
| *Reg3g* | F：CAGACAAGATGCTTCCCCGT |
|  | R：GCAACTTCACCTTGCACCTG |

| *Reg3b* | F：CCCAGGCTTATGGCTCCTAC |
| --- | --- |
|  | R：ATGGAGCCCAATCCAAGTGT |
| *Saa3* | F：AGCCAAAGATGGGTCCAGTT |
|  | R：TCAGAGTAGGCTCGCCACAT |
| *Saa1* | F：ACACTGACATGAAGGAAGCTAAC |
|  | R：CCTCTGCCGAAGAATTCCTGA |
| *Ang4* | F：ACTCTGGCTCAGAATGAAAGGT |
|  | R：GACATCTTTGCAAGGCGAGG |
| *Il22* | F：GGCCAGCCTTGCAGATAACA |
|  | R：GCTGATGTGACAGGAGCTGA |
| *Actb* | F：CTAAGGCCAACCGTGAAAAG |
|  | R：ACCAGAGGCATACAGGGACA |
